# Homeostasis Imbalance of Microglia and Astrocytes Leads to Alteration in the Metabolites of the Kynurenine Pathway in LPS-Induced Depressive-Like Mice

**DOI:** 10.3390/ijms21041460

**Published:** 2020-02-21

**Authors:** Xue Tao, Mingzhu Yan, Lisha Wang, Yunfeng Zhou, Zhi Wang, Tianji Xia, Xinmin Liu, Ruile Pan, Qi Chang

**Affiliations:** Institute of Medicinal Plant Development, Chinese Academy of Medical Sciences and Peking Union Medical College, Beijing 100193, China

**Keywords:** lipopolysaccharide, depression, kynurenine pathway, microglia, astrocytes

## Abstract

In the pathology-oriented study of depression, inflammation hypothesis has received increasing attention for recent years. To mimic the depressive state caused by inflammation, rodents injected intraperitoneally with lipopolysaccharide (LPS) are usually used to stimulate an immune response. However, the dose of LPS that causes depressive-like behavior varies widely across many literatures. Previous study has uncovered the non-linearity in the dose-effect relationship for the depressive-like behavior induced by LPS administration, while the reason for this is still unclear. The present study aims to investigate the underlying mechanisms of this non-linear dose-dependent relationship. Four groups of mice were injected intraperitoneally with different doses of LPS (0, 0.32, 0.8, and 2 mg/kg). The tail suspension test was conducted to evaluate the depressive-like behavior within 23–25 h after the LPS administration. The neuroplasticity was assessed by the levels of related proteins, TrkB and PSD-95, and by the quantification of neurons using Nissl staining. The levels of the two metabolites of the kynurenine (KYN) pathway, 3-hydroxykynurenine (3-HK) and kynurenic acid (KYNA), in the brain were analyzed by LC-MS/MS. Activation of microglia and astrocytes in the brain were also determined by immunohistochemistry and western blotting, respectively. The results showed that, compared with the control group, the mice in the 0.8 mg/kg LPS-treated group exhibited a remarkable increase of immobility time in the tail suspension test. The neuroplasticity of mice in the 0.8 mg/kg LPS-treated group was also significantly reduced. The neurotoxic metabolite, 3-HK, was accumulated significantly in the hippocampus of the 0.8 mg/kg LPS-treated mice. Surprisingly, the 2 mg/kg LPS-treated mice did not exhibit a remarkable change of 3-HK but expressed increased KYNA significantly, which is neuroprotective. Furthermore, the activation of microglia and astrocytes, which were recognized as the primary source of 3-HK and KYNA, respectively, corresponded to the content of these two metabolites of the KYN pathway in each group. Consequently, it was speculated that the homeostasis of different glial cells could lead to a non-linear dose-dependent behavior by regulating the KYN pathway in the LPS-induced depressive-like mice.

## 1. Introduction

Depression is a prevalent psychiatric illness, afflicting patients physically and mentally [1]. By the year 2030, depression is predicted to be the second leading cause of the total global burden of disease [2]. Whereas, pathophysiological causes of this disorder remain elusive. Besides most studies focusing on the function of monoamine neurotransmitters, hypothalamic-pituitary-adrenal (HPA) axis, and neurogenesis [3], there is growing evidence to support the crucial role of neuroinflammation in the etiological explanation for depression [4]. 

Lipopolysaccharide (LPS) is a potent activator of the immune system [5]. Intraperitoneal injection of LPS is reported to lead to symptoms including anhedonia and reduced social and exploratory activity in rodents, which are similar to the clinically relevant symptoms of depression in humans. Thus, LPS is commonly used to study inflammation-associated depression [6]. However, the dose of LPS that causes depressive-like behavior varies tremendously in different literatures, which makes it difficult to compare results over research groups. Thus, the relationship between the dose of LPS and depressive-like behavior needs to be further elucidated. Steven et al. [7] assessed the relationship between the dose of LPS and LPS-induced behavioral changes previously. Unanticipatedly, the depressive-like effects assessed by the forced swimming test were not linearly correlated with the dose of LPS or the production of cytokines in the brain in their study. Although the authors attributed this to the occurrence of sickness behaviors (a behavioral state similar to the clinical symptoms of depression), the confounding effects of sickness behavior were speculated to be responsible for the non-linear dose-dependent behavioral changes in that research [7]. Whereas, it is not entirely convincing to attribute the non-linear dose-dependent changes in depressive-like behavior to sickness behavior directly. Additional factors need to be identified to clarify the underlying mechanisms besides the immune system.

In fact, the pathophysiology of depression is associated with dysfunctions of many systems in the body, including the immune system, monoaminergic system, and glutaminergic system. One potential intersection point of these three systems is the kynurenine (KYN) pathway, of which neuroactive products are involved in the interface between the immune response and serotoninergic neurotransmission via catabolism of tryptophan (Trp) to KYN, ultimately altering downstream synaptic glutamate neurotransmission [8]. Furthermore, a rate-limiting enzyme in the KYN pathway, indoleamine 2, 3-dioxygenase (IDO), has been implicated as a critical molecular mediator of inflammation-induced depressive-like behavior [9].

In the brain, KYN can be distinctively processed by either microglia or astrocytes to produce different neuroactive compounds, such as 3-hydroxykynurenine (3-HK) and kynurenic acid (KYNA). Basically, KYNA can be formed mainly in astrocytes [10], which is neuroprotective via its ability to clear glutamate spillover, while 3-HK can be produced mostly in microglia [11] and exerts powerful excitotoxic effects via potentiating the activation of the NR2B N-methyl-D-aspartate receptor [12]. Evidence suggests that the neuroprotective/neurotoxic balance of the KYN pathway can be altered by the immune response in the central nervous system (CNS) directly or indirectly [13].

Therefore, we hypothesized that the non-linearity in the dose-effect relationship for the depressive-like behavior induced by intraperitoneal injection (i.p.) of LPS might be due to modification of downstream metabolites of the KYN pathway caused by the homeostasis fluctuations of astrocytes and microglia, which led to the further changes in the CNS. The present study was aimed to evaluate the effect of different doses of LPS on depressive-like behaviors, neuroplasticity, the immune system, and KYN pathway in the brain of mice.

## 2. Results

### 2.1. Effects of Different Doses of LPS on the Depressive-Like Behavior of Mice

**Figure 1:**
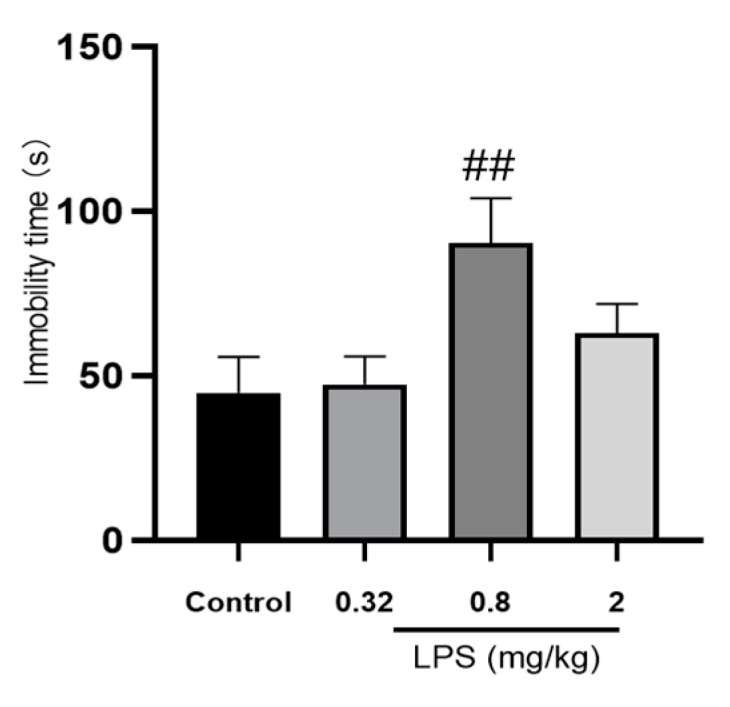
Effects of different doses of LPS (i.p.) on the immobility time of mice in the tail suspension test (TST). The graph is plotted as mean ± SEM (*n* = 12 for each group). Data were analyzed by the Mann–Whitney U test. *^##^ p* < 0.01 compared to the control group.

The effects of different doses of LPS on the depressive-like behavior of mice were assessed by the immobility time in the tail suspension test (TST). As shown in Figure 1, mice treated with 0.8 mg/kg LPS, but not 0.32 or 2 mg/kg, showed a significantly increased immobility time compared to the control group (*p* < 0.05). Unpredictably, the immobility time of 2 mg/kg LPS-treated mice in the TST was only slightly increased, without statistical significance compared to the control group. 

### 2.2. Effects of Different Doses of LPS on Brain Neuroplasticity of Mice

**Figure 2:**
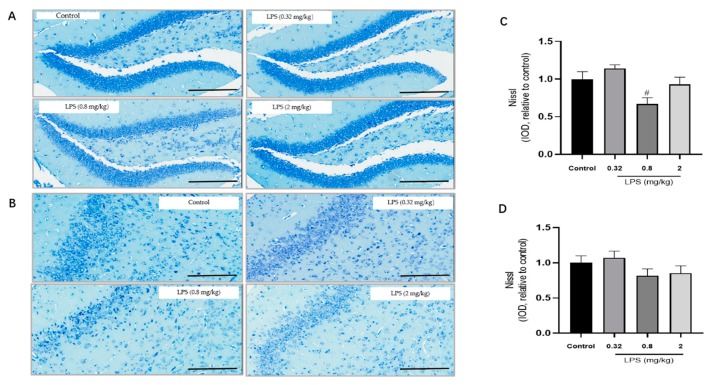

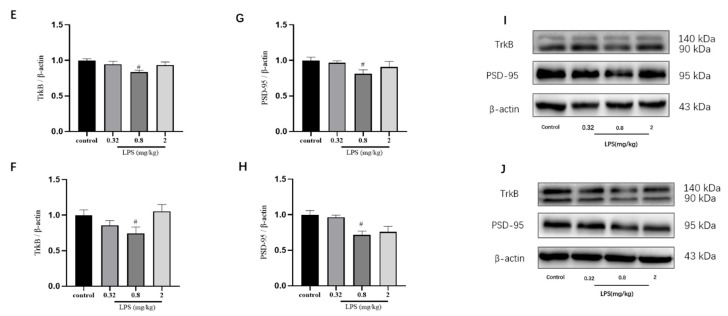
Effects of different doses of lipopolysaccharide (LPS) (i.p.) on brain neuroplasticity of mice. Representative micrographs of Nissl staining in the hippocampus (**A**) and prefrontal cortex (**B**). The scale bar represents 200 μm in the image. Quantification of integrated optical density (IOD) of Nissl bodies in the hippocampus (**C**) and prefrontal cortex (**D**) (*n* = 3 for each group). Representative western blot analysis of TrkB and PSD-95 in the hippocampus (**E**,**G**) and cortex (**F**,**H**) (*n* = 4 for each group). Representative protein bands for the hippocampus (**I**) and cortex (**J**). Graphs are plotted as mean ± SEM. Data were analyzed by the Mann–Whitney U test. *^#^ p* < 0.05 compared to the control group.

To examine whether the LPS-induced non-linear dose-dependent behavior was associated with neuroplasticity, the status of neurons in the hippocampus (Figure 2A) and the prefrontal cortex (Figure 2B) of mice was estimated by the Nissl staining. The integrated optical density (IOD) of Nissl bodies was quantified to show the neuronal status (Figure 2C,D). Nissl staining showed that the neuronal cells in the hippocampus and cortex in the 0.8 mg/kg LPS-treated group were loosely arranged or missing, and Nissl bodies were lightly stained or even dissolved compared with that in other groups. Moreover, LPS induced a slight decrease of Nissl bodies in the two brain regions in the 2 mg/kg LPS-treated mice, and no visible changes of Nissl bodies were observed in the 0.32 mg/kg LPS-treated mice. There was no statistical significance for either group. Since Nissl staining might be non-specific to represent neuronal changes, the expression of TrkB and PSD-95 in the hippocampus (Figure 2E,G) and cortex (Figure 2F,H), both associated with neuroplasticity, were also evaluated by the western blotting. The expression levels of the two proteins in both brain regions of mice exposed to 0.8 mg/kg LPS were significantly lower than that in the control group.

### 2.3. Effects of Different Doses of LPS on the Levels of Metabolites of the KYN Pathway in the Brain of Mice

**Figure 3:**
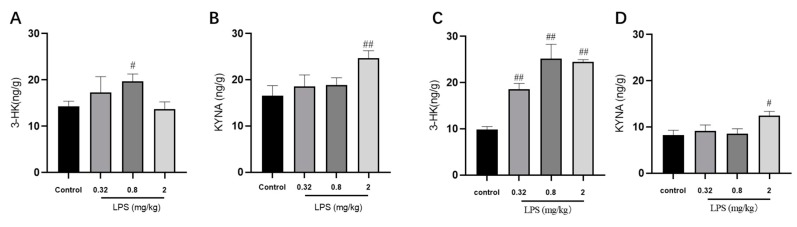
Effects of different doses of LPS (i.p.) on the levels of metabolites of the KYN pathway in the brain of mice. The contents of 3-HK and KYNA in the hippocampus (**A**,**B**) and cortex (**C**,**D**). Graphs are plotted as mean ± SEM (*n* = 7 for each group). Data were analyzed by one-way ANOVA followed by LSD test.*^#^ p* < 0.05, ^*##*^ *p* < 0.01 compared to the control group.

Given the regulatory role of the KYN pathway in the brain development and immune response, the metabolite levels of the KYN pathway in the brain were detected via LC-MS/MS analysis (Figure 3). The results demonstrated that 0.8 mg/kg LPS-treated mice displayed a remarkable increase of 3-HK level. In addition, the level of KYNA, a protective metabolite in the KYN pathway, showed an evident increase in both brain regions of 2 mg/kg LPS-treated animals. 

### 2.4. Effects of Different Doses of LPS on Cytokine mRNA Levels in the Brain of Mice

**Figure 4:**
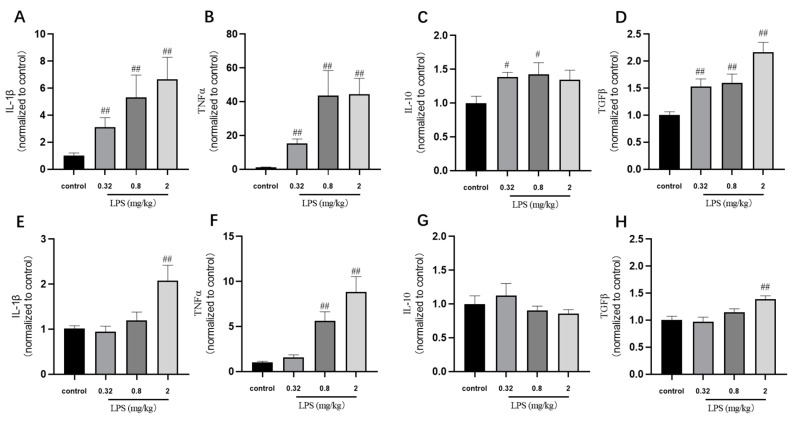
Effects of different doses of LPS on cytokine mRNA levels in the brain of mice. The cytokine IL-1*β*, TNF*α*, TGF*β,* and IL-10 mRNA levels in the hippocampus (**A**–**D**) and cortex (**E**–**H**). Graphs are plotted as mean ± SEM (*n* = 6 for each group). Data were analyzed by one-way ANOVA followed by LSD test. *^#^ p* < 0.05, *^##^ p* < 0.01 compared to the control group.

Many in vivo studies examining the impact of LPS use the endpoint measure of cytokine expression. In our study, the cytokine IL-1*β*, TNF*α*, TGF*β,* and IL-10 mRNA levels in the brain of mice were detected by RT-PCR (Figure 4). The results showed that the levels of cytokine were correlated with the LPS doses basically. The TNFα mRNA levels in both brain regions of mice treated with 0.32, 0.8, and 2 mg/kg LPS were significantly higher than that in the control group (*p* < 0.01). The mRNA levels of IL-1*β*, IL-10, and TGF*β* mRNA in the hippocampus of mice treated with different doses of LPS were similar to the changes of that of TNF*α*. The mice exposed to 2 mg/kg LPS expressed significant increased IL-1*β* and TGF*β* mRNA levels in the cortex (*p* < 0.01), while the 0.32 mg/kg and 0.8 mg/kg LPS-treated group showed a slight increase of these two mRNA levels in the cortex. Not consistent with the behavioral results, these cytokine mRNA levels in the hippocampus and cortex were mostly dose-dependent.

### 2.5. Effects of Different Doses of LPS on the Status of Microglia and Astrocytes in the Brain of Mice

**Figure 5:**
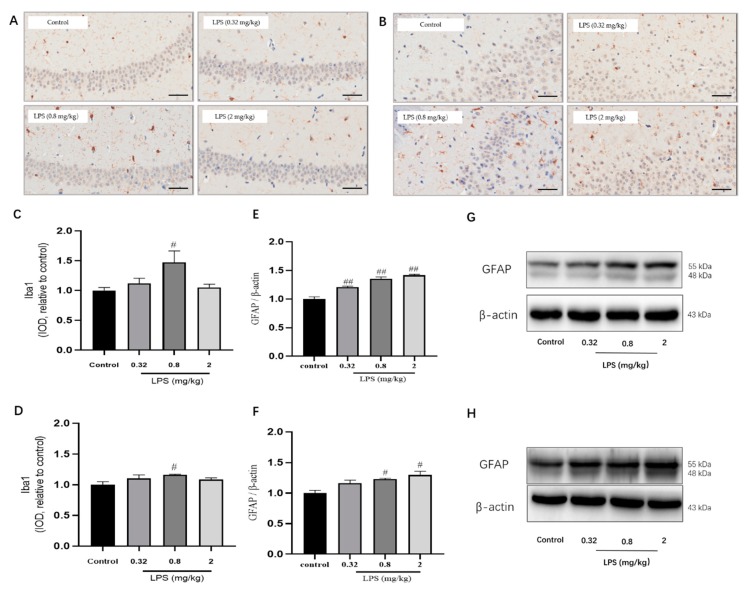
Effects of different doses of LPS (i.p.) on the status of microglia and astrocytes in the brain of mice. Representative micrographs of immunohistochemistry of Iba1 (brown) of microglia in the hippocampus (**A**) and prefrontal cortex (**B**). The scale bar represents 50 μm in these images. Quantification of IOD of microglia in the hippocampus (**C**) and prefrontal cortex (**D**) (*n* = 3 for each group). Representative protein bands of GFAP, a marker for astrocytes, in the hippocampus (**E**,**G**) and cortex (**F**,**H**). Graphs were plotted as mean ± SEM. Data were analyzed by the Mann–Whitney U test. *^#^ p* < 0.05 compared to the control group, *^##^ p* < 0.01 compared to the control group.

Microglia and Astrocytes are two abundant glial cell types in the mammalian CNS. They cannot only secrete cytokines for an immune response but also play differential roles in the process of the KYN pathway. The status of microglia in the hippocampus (Figure 5A,C) and the prefrontal cortex (Figure 5B,D) was determined by immunohistochemistry using the Iba1 marker. The results showed that the IOD of microglia in the hippocampus and cortex of 0.8 mg/kg LPS-treated mice were both remarkably higher than that of the control group (*p* < 0.05), whereas the other two LPS-treated groups showed no statistical significance. The status of astrocytes was detected by glial fibrillary acidic protein (GFAP) using western blotting, and the results of GFAP showed a dose-dependent activation of astrocytes (Figure 5E,F).

## 3. Discussion

The present study is the first to demonstrate that the non-linear dose-dependent depressive-like behavior induced by LPS is caused due to the changes in the KYN pathway regulated by the homeostasis imbalance of microglia and astrocytes, which further affects the neuroplasticity.

A dose range of LPS at 0.32–2 mg/kg was selected based on a previous study [7]. The medium dose was set as 0.8 mg/kg in this study because it was used more frequently in a considerable number of studies [14]. The other two doses of LPS were 0.32 and 2 mg/kg, which were 2.5 folds lower and higher than 0.8 mg/kg, respectively. In the TST, a significant prolonged immobility time in the 0.8 mg/kg LPS-treated group was observed, while a similar effect was not found in the other two groups. For excluding the false positives caused by changes in motor activity, we also evaluated spontaneous motor activity for the different doses of LPS used in this study. Compared with the control group, the mice in each LPS-treated group moved slightly less in the open field test, but there was no significant difference (data not shown). Interestingly, a non-linear relationship between dose and behavioral changes showed in the TST was similar to that observed in a previous study [7]. Because no linear correlation between inflammatory factors and behavioral changes was observed in that study, it was speculated that not the inflammatory factors, but rather the confounding effects of sickness behavior to be responsible for the non-linear dose-dependent behavioral changes. However, this assumption lacks powerful evidence. In this study, we tried to explore the underlying mechanism of this non-dose-dependent behavior beyond inflammatory factors. Neuroplasticity is a fundamental contributor to adaptive functioning [15]. In the clinical neuroscience community, considerable evidences have been demonstrated that dysfunctional neuroplasticity might underlie the pathophysiology of depression, and antidepressants exerted a significant influence on neuroplasticity [16]. PSD-95 and TrkB, a major synaptic scaffolding protein [17] and the receptor for brain derived neurotrophic factor [18], respectively, play a crucial role in the enhancement of neuroplasticity. For the 0.8 mg/kg LPS-treated animals, the significant decrease of PSD-95 and TrkB expressions were observed. In tune with that, the results of Nissl staining in our study showed that the neuronal cells in the hippocampus and cortex were loosely arranged and lightly stained in the 0.8 mg/kg LPS-treated mice. Interestingly, PSD-95 and TrkB levels, as well as the morphology of the neuronal cell, did not decline significantly in the other two LPS-treated groups. As discussed above, 0.8 mg/kg LPS-treated mice indicated a remarkable dysfunction of neuroplasticity.

It is best known that the immune system is supposed to express pro- or anti-inflammatory cytokines [19] once triggered by internal and external threats [20]. In our study, the mRNA expressions of IL-1 *β*, TNF*α*, TGF*β*, and IL-10 in the hippocampus and cortex of mice were determined by RT-PCR. In accordance with the findings of Steven et al. [7], the cytokine levels in our study were positively correlated with the dose of LPS, whereas they were not completely consistent with behavioral changes. In addition, mice treated with 0.32 mg/kg LPS showed a significant increased expression of these cytokines in the hippocampus compared with those in the normal group, while there were no significant changes in the cortex. This might be related to differences in the sensitivity of the two brain regions to inflammatory cytokines [21]. Nevertheless, it could be indicated that the changes in cytokines in CNS were insufficient to account for the non-linear dose-dependent behavioral changes.

Generally, the KYN pathway is indicated to play a crucial role in integrating the nervous system and immune system [22,23]. As one of the main metabolic pathways of Trp, the rate-limiting step of the KYN pathway in the brain is the conversion of Trp to KYN [9,22]. In patients with depression, the ratio of kynurenine to tryptophan in blood is significantly enhanced and correlates with anxiety and cognitive deficits [24]. Interestingly, the conversion of Trp to kynurenine is mediated by IDO enzymes, of which the activity can be dramatically inducible by immune stimuli [25]. The causative role of the KYN pathway in the LPS-induced depressive-like behavior has been indicated in a previous study [9]. It was reported that blockade of IDO activation either indirectly with the anti-inflammatory tetracycline derivative minocycline or directly with the IDO antagonist 1-methyltryptophan (1-MT), prevented development of depressive-like behavior. Moreover, the administration of kynurenine to naïve mice was demonstrated to dose-dependently induce depressive-like behavior.

In addition to the essential role of IDO at the initial step, the KYN pathway can yield metabolites with neuroprotective and neurotoxic properties [26]. Basically, KYN is usually converted to 3-HK and proceeds with the conversion to quinolinic acid (QUIN), both of which are neurotoxic. 3-HK and QUIN are preferred products of the KYN pathway, while the other branch of the pathway, leading to the production of KYNA, can increase under Trp or KYN loading [27]. In our study, the expression of 3-HK was significantly increased in the 0.8 mg/kg LPS-treated group compared with the control group, while no significant changes were observed in the groups treated with other doses of LPS. All results above showed that changes in behavior and neuroplasticity in the 0.8 mg/kg LPS-treated group might be directly or indirectly associated with the accumulation of 3-HK in the brain.

Nevertheless, what role does the immune system play in the non-linear dose-dependent behavioral changes of LPS-treated animals? We noticed a significant increase in KYNA levels in the hippocampus and cortex of mice treated with LPS at a dose of 2 mg/kg, which is significantly different from the other two treated groups. And a significant increase in 3-HK levels was observed in the hippocampus and cortex of mice treated with LPS at a dose of 0.8 mg/kg. KYNA and 3-HK are best known for the downstream of the KYN pathway. The latter is neurotoxic, as mentioned above, while the former is neuroprotective as binding at the NR2B N-methyl-D-aspartate receptor (NMDAR) to clear glutamate spillover in the CNS [13]. Although a significant increase of 3-HK level was demonstrated in the cortex of mice treated with not only 0.8 mg/kg of LPS but also with 0.32 and 2 mg/kg, it might be due to the activation of microglia in the cortex of all LPS-treated groups. The roles that 3-HK and KYNA played depended on the change in the equilibrium state between them. It was speculated that the attenuation of depressive-like behavior in the 2 mg/kg LPS-treated group might be attributed to the increase of KYNA, which partially counteracted neurotoxic effects of 3-HK. So how does the immune system regulate the concentration of these two metabolites? Notably, the production of 3-HK mainly occurred in microglia [23], while astrocytes were the primary source of KYNA [10]. Subsequently, the status of microglia and astrocytes in the hippocampus and cortex were measured. The activation of microglia in the 2 mg/kg LPS-treated group was found to be lower than that in the 0.8 mg/kg LPS-treated group, while the trend of astrocytes activation was reversed between the two groups. The results indicated that microglia activation might not be simply affected by the dose of LPS administration. After activation, different types of glia have different responses, including secreting toxic factors to kill microorganisms, secreting anti-inflammatory cytokines to reduce stress, and so on [28]. A previous work indicated that an anti-inflammatory factor produced by microglia, IL-10, can stimulate astrocytic TGF*β*, providing negative feedback on microglial activation [29], which affected the homeostasis of glia cells. Consequently, this homeostasis fluctuation of different glia cells might directly or indirectly influence their neuroprotective or neurotoxic effects. Another study has demonstrated that astrocytes alone were protective, but the production of a large amount of KYN in astrocytes might be secondarily metabolized by adjacent microglia, inducing neurotoxicity indirectly [10]. 

In addition, there are some limitations in this study that could be addressed in future research. First, CD68/ED1 can be used as the microglia marker to investigate the status of activated microglia. Second, cell and animal experiments with related inhibitors need to be conducted to further elucidate the causal role of homeostasis imbalance of microglia and astrocytes in the alterations of metabolites of the KYN pathway in LPS-induced depressive-like mice. 

Taken together, our present study proposed for the first time that LPS administration could change the levels of the metabolites of the KYN pathway by regulating the homeostasis of different glial cells, and ultimately affect the neuroplasticity of the central nervous system. This study attested to the claims that LPS administration could induce non-linear dose-dependent behavioral changes and was the first to explain this non-linear dose-effect relationship from the alterations of neuroplasticity and inflammatory response. Nevertheless, this work was expected to provide a basis for the establishment of a much more reliable depression model induced by LPS and a powerful physiological basis for the study of inflammation-associated depression.

## 4. Materials and Methods

### 4.1. Animals

Male C57BL/6J mice (7–8 weeks old) were purchased from the Beijing Weitong Lihua Experimental Animal Technology Company. Procedure rooms were maintained at a temperature of 23 ± 1 °C and a humidity of 60% ± 5%. All animals were bred and housed four per cage with standard food and water available ad libitum. They were maintained on a 12 h light/dark cycle (lights on at 08:30 a.m.). All animal care and experimental protocols were approved by the Committee on Animal Care and Use of the Institution of Medical Plant Development, Chinese Academy of Medical Sciences and Peking Union Medical College (SLXD-20180605006, 5 June 2018).

### 4.2. Drugs and Reagents

Lipopolysaccharide (LPS) from *Escherichia coli* (serotype O55:B5) was purchased from Sigma-Aldrich and freshly dissolved in sterile saline prior to injection. 5-HT, 5-HIAA, TRP, KYNA, 3-HK, and 4-acetamidophenol (internal standard, IS) were obtained from Sigma-Aldrich (St. Louis, MO, USA). Acetonitrile and formic acid (mass-spectrometry grade) were provided by Fisher Co., Ltd. (Waltham, MA, USA). Trifluoroacetic acid (TFA) was purchased from Dikma (Lake Forest, CA, USA).

### 4.3. Experimental Design

After 7-day acclimation, the mice were randomly divided into four groups (*n* = 12) by body weight: control group (saline), 0.32 mg/kg LPS-treated group, 0.8 mg/kg LPS-treated group, and 2 mg/kg LPS-treated group. The tail suspension test (TST) was conducted within 23–25 h after the i.p. injection of LPS. After the TST, the mice were sacrificed by decapitation. The cerebral cortex and hippocampus from nine mice randomly selected in each group were rapidly separated and stored at −80 °C before further analysis. The remaining three mice in each group were perfused transcardially with saline, followed by cold 4% paraformaldehyde (PFA) in PBS. The brains were excised and fixed in 4% PFA for 24 h. Following paraffin embedding, serial 4 μm-thick coronal sections were performed with Nissl or immunohistochemistry (IHC) staining by standard protocols.

### 4.4. Tail Suspension Test

The TST was conducted according to our previously reported method [30]. In brief, each mouse was suspended with its tail fixed by adhesive tape (approximately 1 cm from the tip of tail). All of the mice were suspended for 6 min, and the immobility time during the last 4 min was recorded by video tracking software. The mice were regarded as immobile only when they hung passively and were completely motionless. Additionally, the instrument and video tracking software are independently developed and patented, but not yet commercialized.

### 4.5. LC-MS/MS Analysis

LC-MS/MS analysis was performed as the method described by Wang et al. [31]. Basically, it was conducted using QTRAP 5500 mass spectrometer (AB SCIEX, Foster City, CA, USA) connected with a SHIMADZU Prominence LC system (Kyoto, Japan), equipped with the Restek Ultra Aqueous C18 column (100 mm × 2.1 mm, 3 µm, Bellefonte, PA, USA). The mobile phase consisted of water containing 0.1% formic acid (A) and acetonitrile (B) at a flow rate of 0.4 mL/min. The gradient elution was programmed as follows: 0–0.5 min, 95% A; 0.5–5 min, 95–20% A; 5–6 min, 20% A; 6–6.1 min, 20–95% A and 6.1–8 min, 95% A. The mass detection was conducted using multiple reaction monitoring (MRM) analysis with the electrospray ionization (ESI) source in positive mode. The MRM transitions were set as follows: m/z 204.8/188.0 (TRP), 189.9/143.9 (KYNA), 225.0/109.8 (3-HK), 177.0/160.0 (5-HT), 192.0/146.0 (5-HIAA), and 152.0/109.9 (IS). Data acquisition and procession were conducted by using the Analyst software version1.6.1 (AB SCIEX, Concord, ON, Canada).

### 4.6. Western Blotting Analysis

The proteins of the hippocampus or cortex were extracted using a proper amount of protein extraction solution containing 1% protease inhibitors and 1% phosphatase inhibitors. Following centrifuging at 20,000 *g* for 20 min, the supernatant of the mixture was collected and quantified by BCA protein assay. Twenty-five microgram of quantified protein was separated by the sodium dodecyl sulfate-polyacrylamide gel electrophoresis (SDS-PAGE) and it was then transferred onto a nitrocellulose membrane. After a 2 h of block in 7.5% milk solution, the membrane was incubated with primary antibodies at 4 °C overnight, including anti-TrkB (1:1000, abcam), anti-GFAP (1:1000, abcam) and anti-PSD-95 (1:1000, abcam), respectively. Subsequently, the membrane was rinsed three times (10min per time) and then incubated with appropriate horseradish peroxidase-linked secondary antibodies (1:5000, ABclonal) at room temperature for another 2 h. Finally, the protein was detected by enhanced chemiluminescence. The grey intensity values of bands were analyzed using Image J 1.46r software (NIH, Bethesda, MD, USA).

### 4.7. Quantitative Reverse Transcription Polymerase Chain Reaction Analysis (RT-PCR)

Total RNA was extracted from the cortex using TRIzol regent according to the manufacturer’s instructions (TransGen, Beijing, China). Trace contamination of DNA was removed by DNase digestion. The Reverse Transcription System (Roche) was used for the first strand cDNA synthesis. Quantitation of all gene transcripts was performed in IQ 5.0 ABI 7500 (Bio-Rad, Hercules, CA, USA) using Top Green qPCR SuperMix (TransGen, Beijing, China) The sequences of primers were as follows: IL-1*β* forward 5′-CACTACAGGCTCCGATGAACAAC-3′, IL-1*β* reverse 5′-TGTCGTTGCTTGGTTCTCCTTGTAC-3′, TNF*α* forward 5′-ATGTCTCAGCCTCTTCTCATTC-3′ and TNF*α* reverse 5′-GCTTGTCACTCGAATTTTGAGA-3′, IL-10 forward 5′-TCCCTGGGTGAGAAGCTGAAGAC-3′, IL-10 reverse 5′-CACCTGCTCCACTGCCTTGC-3′, TGF*β* forward 5′-ACCGCAACAACGCCATCTATGAG-3′ and TGF*β* reverse 5′-GGCACTGCTTCCCGAATGTCTG-3′. Forty cycles of the profile were run. Amplicon size and specificity were confirmed by the melting curve analysis. Relative mRNA expression of the target gene was defined as 2^−ΔΔCt^.

### 4.8. Nissl Staining

Nissl staining was carried out as described before [32]. Brain sections were dehydrated in ascending grades of ethanol, immersed in xylene, rehydrated in descending grades of ethanol, and hydrated in distilled water. Subsequently, the sections were stained with toluidine blue, dehydrated in ethanol, and cleared in xylene before coverslipped with Permount mounting medium.

### 4.9. Immunohistochemistry

IHC analysis was conducted as Zhu et al. described before with a slight modification [33]. Briefly, free-floating brain sections were rinsed in 0.1 M PBS (pH = 7.4) for 3 times following antigen retrieval and incubated in 3% H_2_O_2_ for 25 min. Then they were blocked for non-specific antigen binding using 3% BSA for 30 min at room temperature, which was followed by an overnight incubation with primary antibody against Iba1 (1:200, Abcam) at 4 °C. Sections were then rinsed in 0.1 M PBS for 3 times and incubated with horseradish peroxidase-linked anti-mouse IgG for 50 min at room temperature. One set with hematoxylin counterstaining was used for cellular location, which was performed according to the manufacturer’s instructions (Servicebio, Wuhan, China). Sections were then rinsed, dehydrated in 75%, 85%, and 100% ethyl alcohol, cleared in xylene and coverslipped with Permount (Servicebio, Wuhan, China) in a fume hood.

### 4.10. Unbiased Estimation of the Status of Neurons and Microglia

The images of sections were acquired at 20× (for Nissl staining) or 40× (for IHC) magnifications utilizing the Aperio CS2 system (Leica Biosystems Imaging, Buffalo Grove, IL, USA). With a threshold that discriminated staining from the background best, the integrated optical density (IOD) above the threshold was calculated for each image with equal size by ImageJ software (NIH, Bethesda, MD, USA).

### 4.11. Statistical Analysis

Data were expressed as mean ± SEM. One-way ANOVA followed by post hoc LSD test was performed to determine the significance for multiple comparisons when the data meet normal distribution. When the data did not meet the requirements of normal distribution, Mann–Whitney U test was conducted. Significance was set at *p* < 0.05. Calculations were performed using SPSS statistical software (version 17.0) (IBM, Armonk, NY, USA).
